# Supplementary material for: Loss of GFAT-1 feedback regulation activates the hexosamine pathway that modulates protein homeostasis
Source: Nat Commun. 2020 Feb 4;11:687. doi: 10.1038/s41467-020-14524-5 (PMC7000685; doi:10.1038/s41467-020-14524-5)
Supplement: Supplementary file 1 — Supplementary Information [file 41467_2020_14524_MOESM1_ESM.pdf]

Supplementary Information

Loss of GFAT-1 feedback regulation activates the hexosamine pathway that modulates protein homeostasis

Sabine Ruegenberg, Moritz Horn et al.

Fig. 1 Supp: Structure of human GFAT-1, the key enzyme of the hexosamine pathway

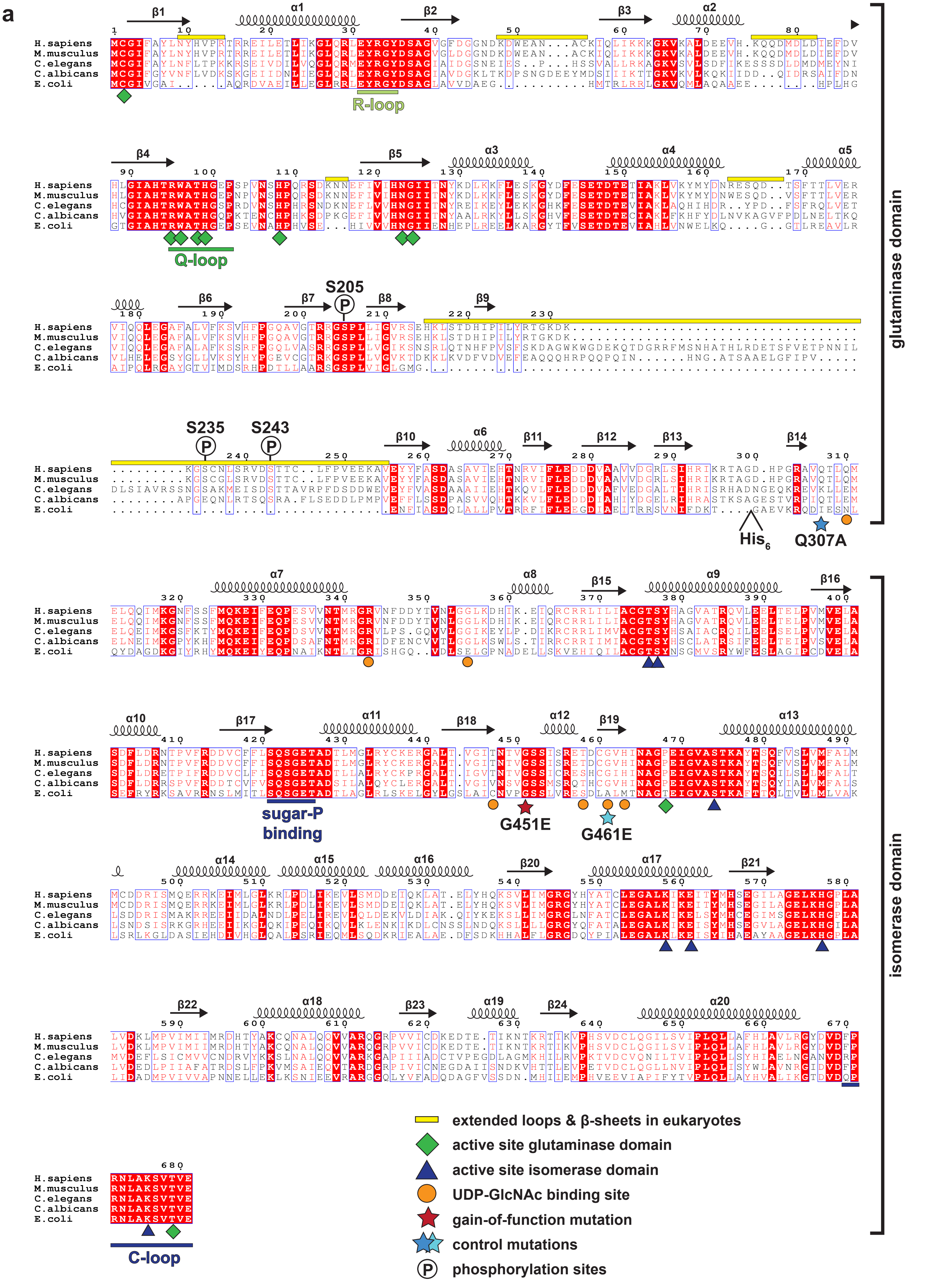

Fig. 1 Supp: Structure of human GFAT-1, the key enzyme of the hexosamine pathway

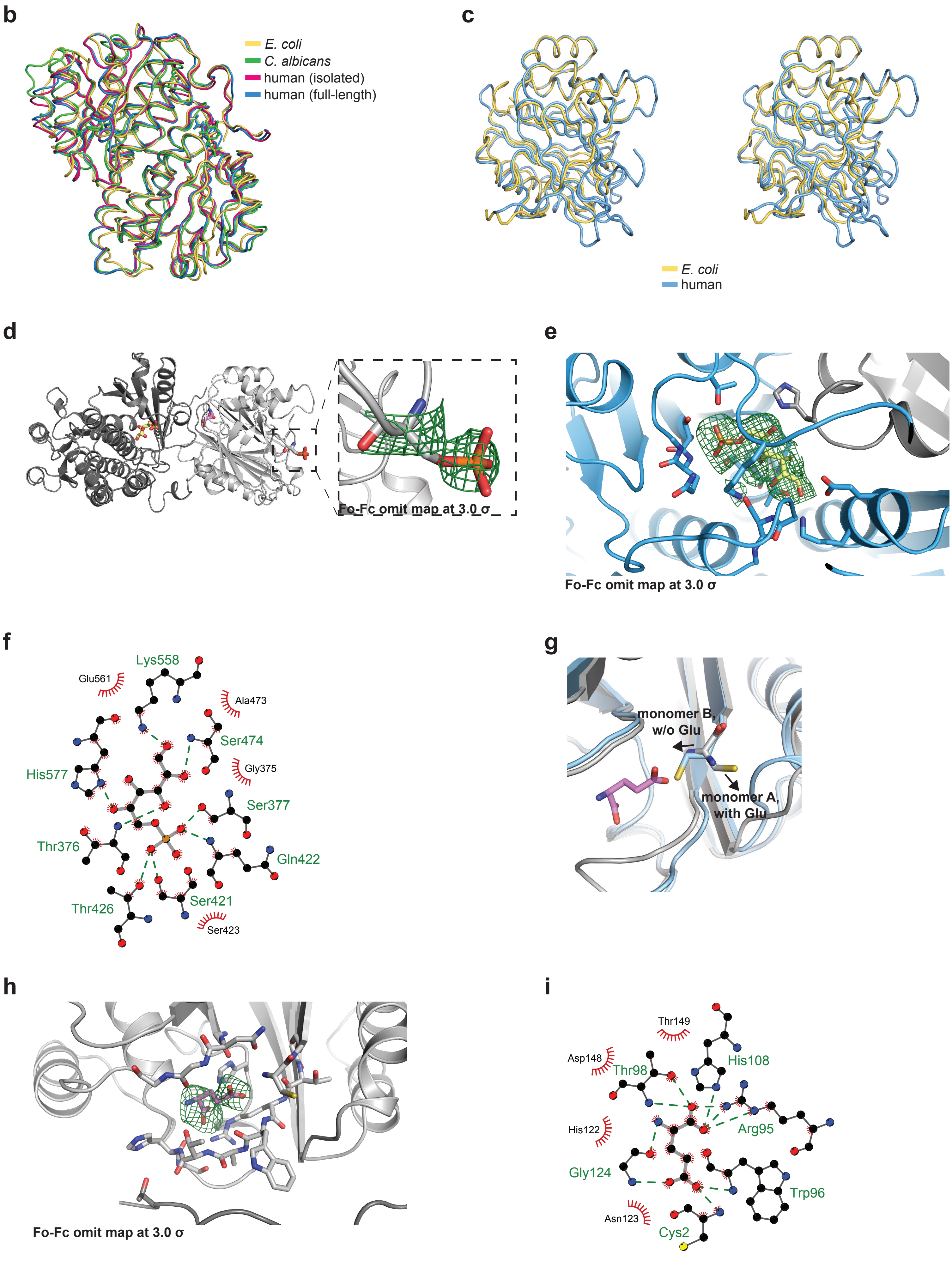

Supplementary Fig. 1: Structure of human GFAT-1, the key enzyme of the hexosamine pathway.

a, Protein sequence alignment of GFAT-1. Red boxes indicate identical residues, red letters indicate similar residues. Secondary structure elements are annotated. Extended loops and β-sheets in the human enzyme compared to the bacterial enzymes are highlighted with yellow bars. **b**, Superposition of *E. coli* (yellow, PDB: 2J6H), *C. albicans* (green, PDB: 2PUW), isolated human isomerase domain (magenta, PDB: 2ZJ3), and full-length human GFAT-1 isomerase domain (marine) with RMSDs of 1.68 Å, 1.38 Å and 0.44 Å respectively over 344 to 360 main chain residues, ribbon representations. **c**, Stereo image of the superposition of *E. coli* (yellow, PDB: 2J6H) and human GFAT-1 glutaminase domain (blue) with RMSD of 2.68 Å over 232 main chain residues, ribbon representations. **d**, Position of phosphorylated Ser243. Close-up of the electron density at Ser243. The Fo-Fc omit map (green) has a contour level of 3.0 RMSD. **e**, **h**, Fo-Fc omit maps (green) of the active sites (**e**, isomerase; **h**, glutaminase) of human GFAT-1 at a contour level of 3.0 RMSD. The protein is in cartoon representation, residues involved in substrate binding or catalysis are highlighted as sticks. **f**, **i**, 2D ligand-protein interaction diagrams of Glc6P (**f**) and L-Glu (**i**) with the respective active site. Ligand bonds are colored in gray, amino acid side chain bonds in black. Green dashed lines indicate hydrogen bonds and red spiked arcs present residues additionally stabilizing the ligand. **g**, Superposition of L-Glu (violet sticks) binding site in the presence (gray) and absence (blue) of L-Glu. L-Glu binding influences the orientation of Cys2 (gray/blue sticks).

Fig. 2 Supp: GFAT-1 forms an asymmetric dimer

a

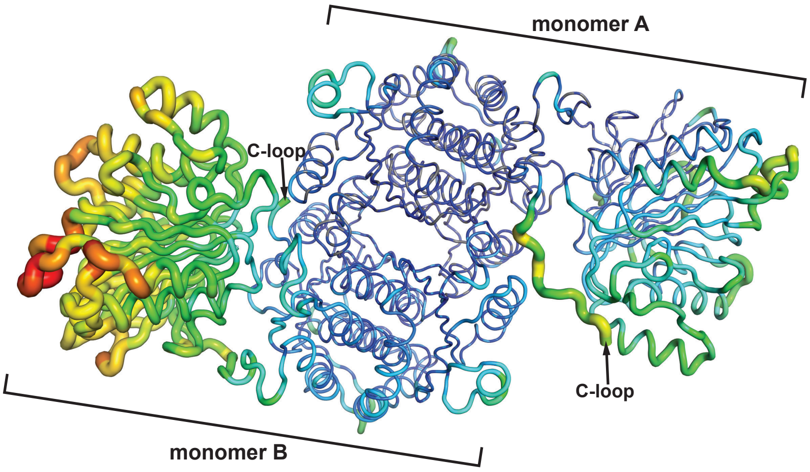

b

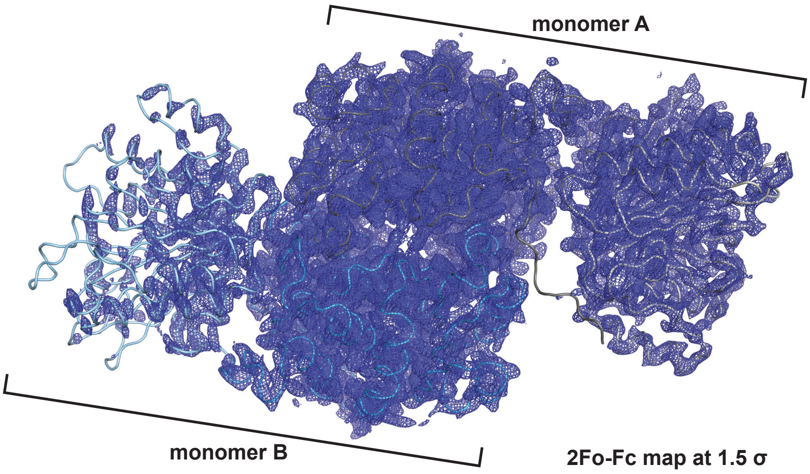

c

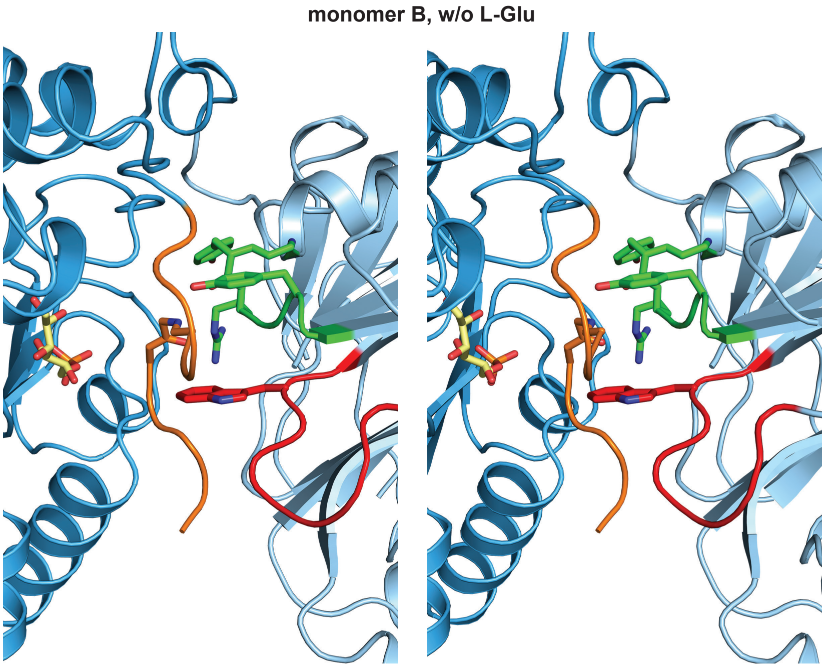

Fig. 2 Supp: GFAT-1 forms an asymmetric dimer

d

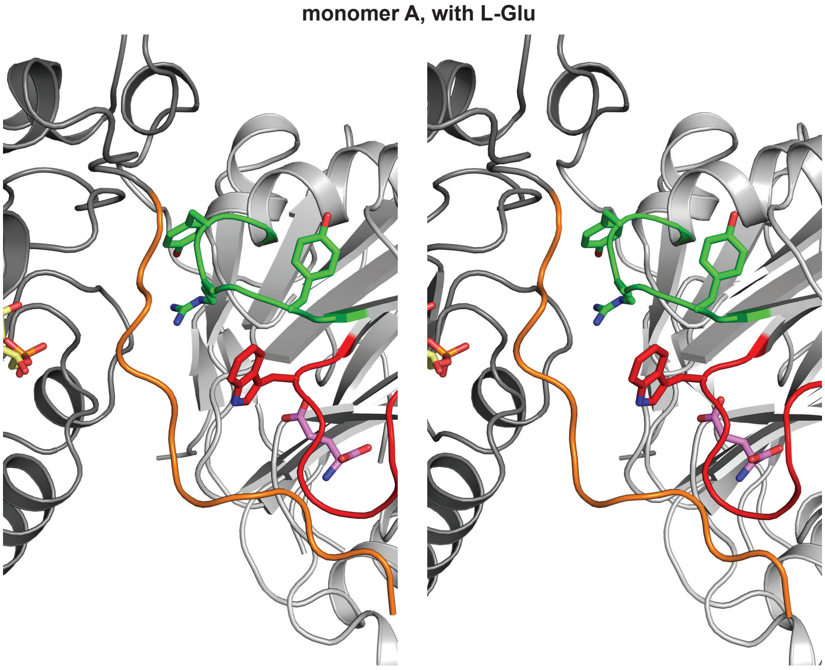

e

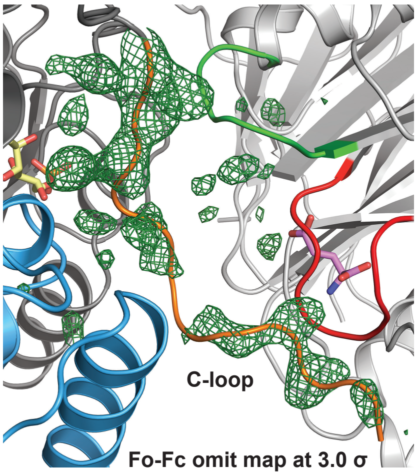

f

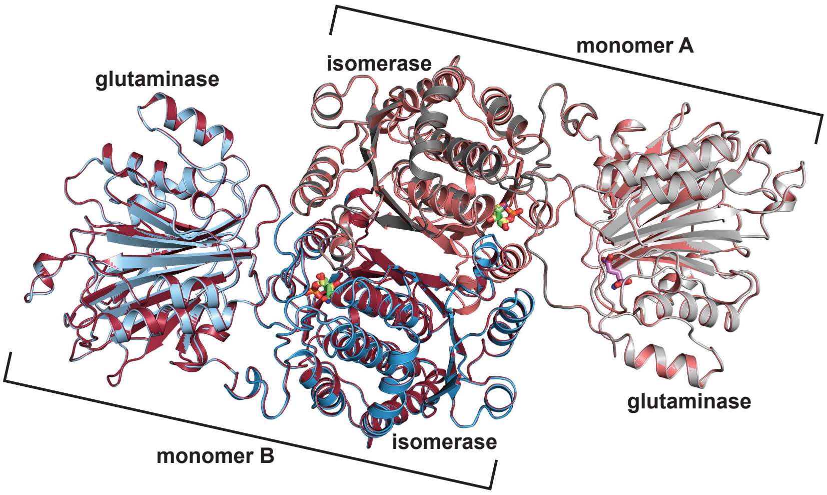

g

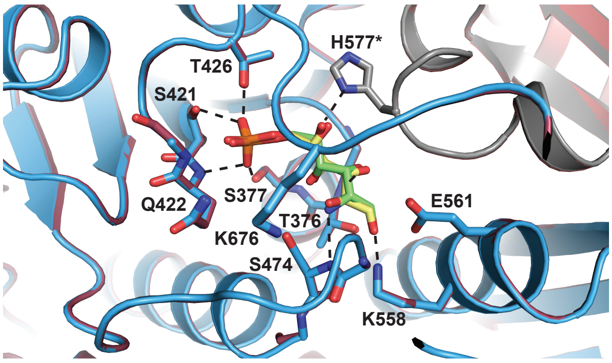

h

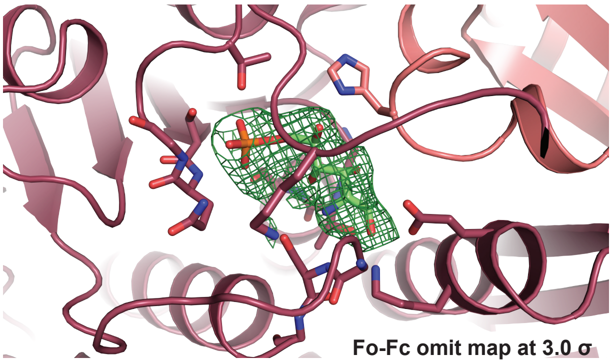

Supplementary Fig. 2: GFAT-1 forms an asymmetric dimer.

a, Representation of B-factors in wild type human GFAT-1 as putty cartoon. Colored from low to high values (38 to 256 $Å^2$, blue to red). **b**, Electron density of wild type human GFAT-1. GFAT-1 is presented as ribbon representation and colored in gray (monomer A) and blue (monomer B). The 2Fo-Fc map is colored dark blue and its contour level is at 1.5 RMSD. **c-e**, Comparison of the C-loop orientation in monomer A and monomer B. GFAT-1 is presented as a cartoon and colored in gray (monomer A) and blue (monomer B). Glc6P (yellow sticks) and L-Glu (violet sticks) are highlighted, as well as important loops: R-loop (green), Q-loop (red), and C-loop (orange). **c**, Stereo image of GFAT-1 monomer B w/o L-Glu. **d**, Stereo image of GFAT-1 monomer A with L-Glu. **e**, Electron density around the C-loop. The Fo-Fc omit map is colored green and its contour level is at 3.0 RMSD. **f**, **g**, Superposition of Glc6P-bound and GlcN6P-bound GFAT-1 with RMSD of 0.14 Å over 1320 main chain residues. Proteins are presented as cartoons. Glc6P-bound GFAT-1 is depicted in gray/blue and GlcN6P-bound GFAT-1 is colored in red. Glc6P (yellow sticks), GlcN6P (green sticks), and L-Glu (violet sticks) are highlighted. **f**, Overall structure of the human GFAT-1 dimer. **g**, Frc6P/GlcN6P-binding site formed by both isomerase domains. Residues involved in binding or catalysis are highlighted as sticks, and dashed lines indicate key interactions. **h**, Fo-Fc omit map (green) of GlcN6P bound to the isomerase active site at a contour level of 3.0 RMSD. The protein is in cartoon representation, residues involved in substrate binding or catalysis are highlighted as sticks.

Fig. 3 Supp: Glutamate determines conformation of Q-loop, R-loop, and C-loop

a

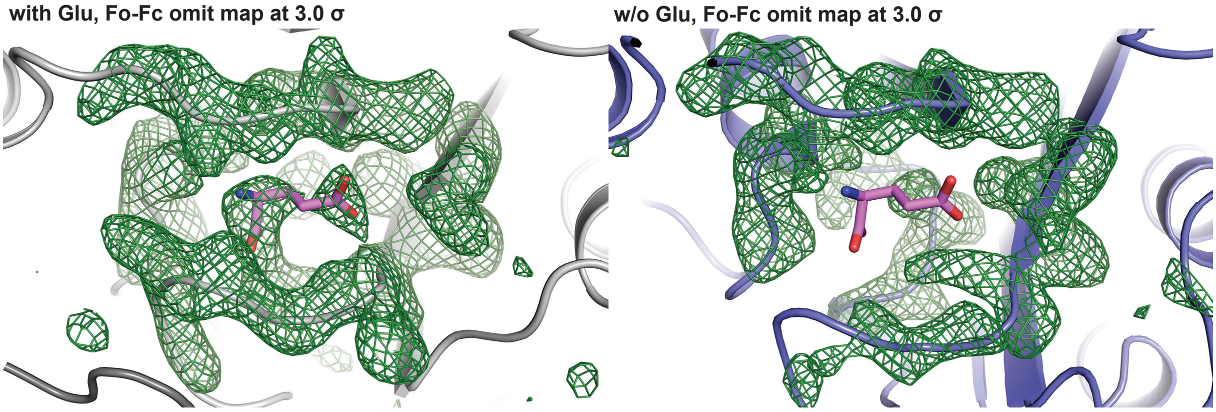

b

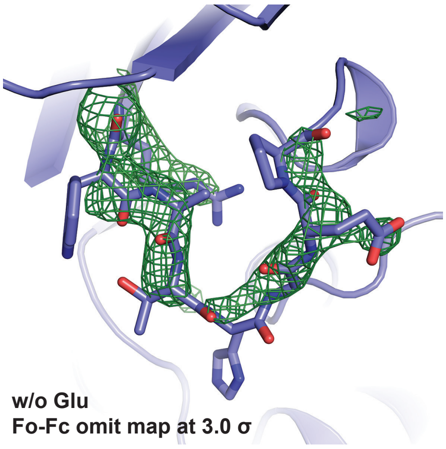

Supplementary Fig. 3: Glutamate determines conformation of Q-loop, R-loop, and C-loop.

a, Electron density around L-Glu at the glutaminase site of GFAT-1 monomer A. Proteins are presented as cartoons. Glu-bound GFAT-1 is depicted in gray and Glu-free GFAT-1 is colored in purple. L-Glu (violet sticks) is highlighted. The Fo-Fc maps omitting 6 Å around L-Glu are colored green and its contour level is at 3.0 RMSD. **b**, Electron density around the Q-loop in Glu-free GFAT-1 monomer A. Glu-free GFAT-1 is presented as cartoon and colored in purple. Side chains of the Q-loop are highlighted as sticks. The Fo-Fc omit map is colored green and its contour level is at 3.0 RMSD.

Fig. 4 Supp: GFAT-1 inhibition by UDP-GlcNAc and UDP-GalNAc

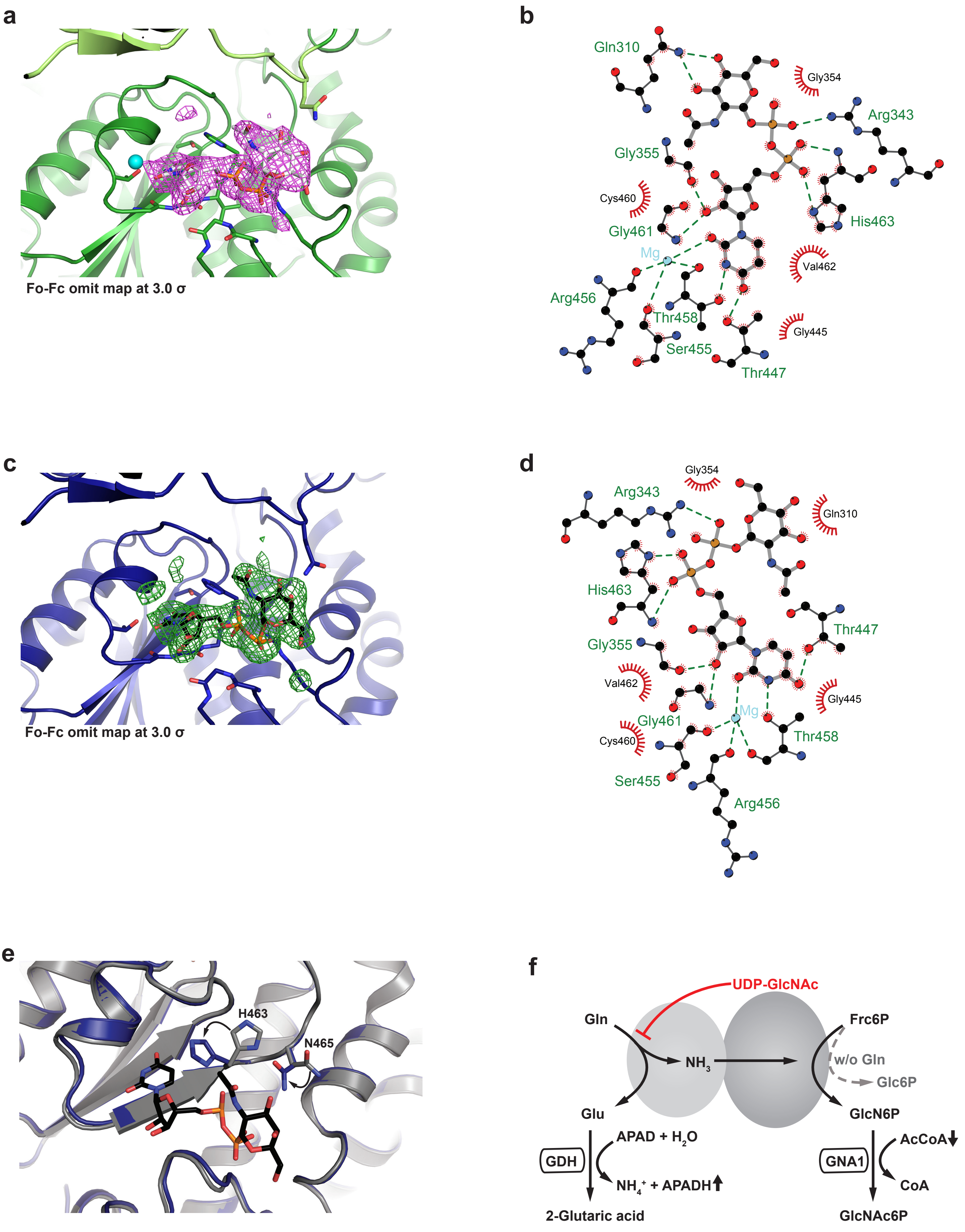

Fig. 4 Supp: GFAT-1 inhibition by UDP-GlcNAc and UDP-GalNAc

g

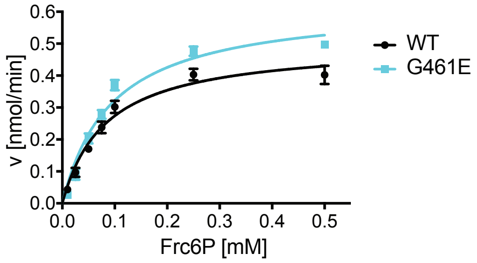

h

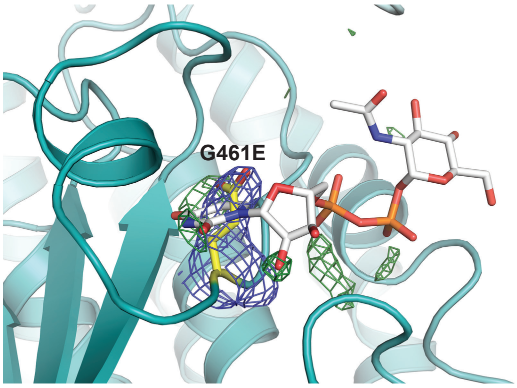

2Fo-Fc map at 1.0 σ, Fo-Fc omit map at 3.0 σ

i

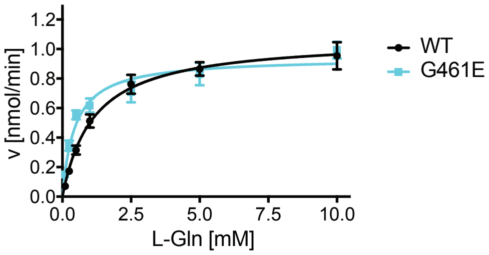

j

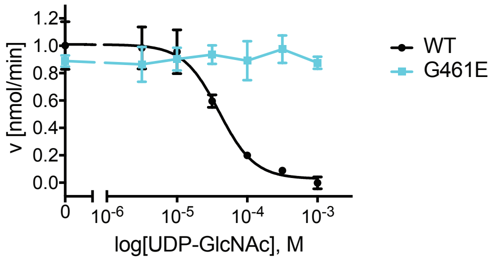

	**UDP-GlcNAc inhibition**
	IC_{50} [μM]
**WT**	49.0 -5.2/+5.8
**G461E**	NA

Supplementary Fig. 4: GFAT-1 inhibition by UDP-GlcNAc and UDP-GalNAc.

a, Fo-Fc omit map (pink) of UDP-GlcNAc binding to human GFAT-1 at a contour level of 3.0 RMSD. UDP-GlcNAc-bound GFAT-1 is presented as a green cartoon. UDP-GlcNAc (white sticks) and residues involved in substrate binding or catalysis are highlighted as sticks. **b**, **d**, 2D ligand-protein interaction diagrams of UDP-GlcNAc (**b**) and UDP-GalNAc (**d**) interacting with GFAT-1. Ligand bonds are colored in gray and amino acid side chain bonds in black. Green dashed lines indicate hydrogen bonds and red spiked arcs present residues additionally stabilizing the ligand. **c**, Fo-Fc omit map (green) of UDP-GalNAc binding to human GFAT-1 at a contour level of 3.0 RMSD. UDP-GalNAc-bound GFAT-1 is presented as a blue cartoon. UDP-GalNAc (black sticks) and residues involved in substrate binding or catalysis are highlighted as sticks. **e**, Locally occurring side chain movements upon UDP-GalNAc binding. Proteins are presented as cartoons. UDP-GalNAc-bound GFAT-1 is depicted in blue and UDP-GalNAc-free GFAT-1 is colored in gray. UDP-GalNAc (black sticks) is highlighted. **f**, Schematic representation of GFAT catalysis, including glutamate dehydrogenase (GDH) and glucosamine 6-phosphate N-acetyltransferase (GNA-1) coupled-enzymatic assays. **g**, Frc6P kinetic of wild type (WT, black circle) and G461E (teal square) GFAT-1 (mean ±SEM, n=5). **h**, GFAT-1 G461E mutation blocks UDP-GlcNAc binding. Close-up of the UDP-GlcNAc binding pocket in the GFAT-1 G461E mutant structure (cyan, cartoon representation). The G461E mutation is highlighted with yellow sticks and the 2Fo-Fc map (blue) of the mutation has a contour level of 1.0 RMSD. UDP-GlcNAc (white sticks) is modeled into the structure by superposition with the wild type UDP-GlcNAc-bound structure. The Fo-Fc omit map (green) after UDP-GlcNAc-soaking of UDP-GlcNAc is shown and has a contour level of 3.0 RMSD. **i**, L-Gln kinetic of wild type (WT, black circle) and G461E (teal square) GFAT-1 (mean ±SEM, n=5). **j**, Representative UDP-GlcNAc inhibition of wild type (black circle) and G461E (teal square) GFAT-1 (mean ±SD, n=3). Table: IC_{50} UDP-GlcNAc values (mean ±SEM, n=3). Source data are provided as a Source Data file.

Fig. 5 Supp: GFAT-1 gain-of-function mutation perturbs UDP-GlcNAc inhibition

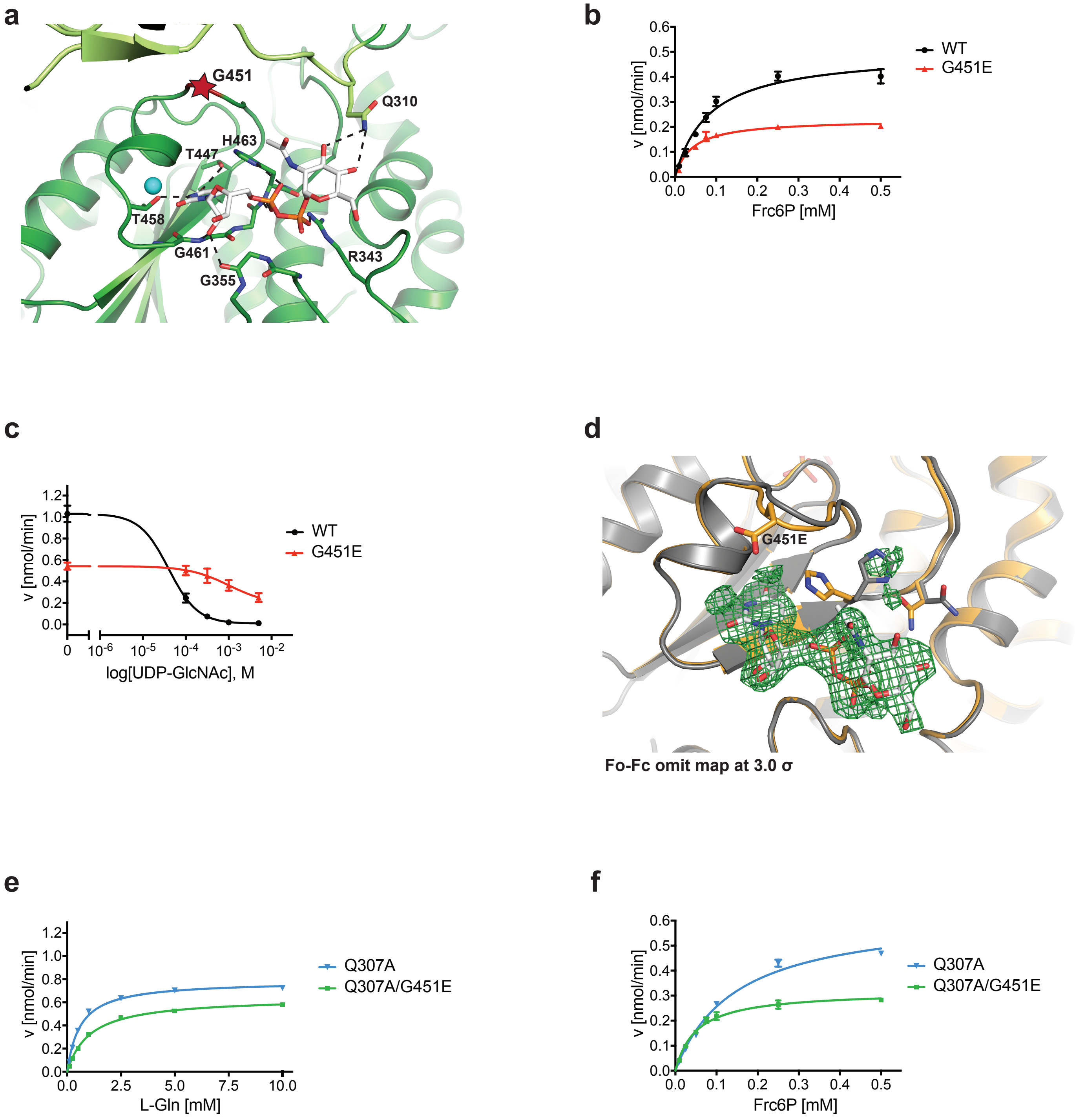

Supplementary Fig. 5: GFAT-1 gain-of-function mutation perturbs UDP-GlcNAc inhibition.

a, Cartoon representation of wild type UDP-GlcNAc-bound GFAT-1 (green). UDP-GlcNAc (white sticks), Mg^{2+} (cyan sphere), and the position of G451 (red star) are highlighted. **b**, Frc6P kinetic of wild type (WT, black circle) and G451E (red triangle) GFAT-1 (mean ±SEM, n=5). **c**, Representative UDP-GlcNAc inhibition of wild type (WT, black circle) and G451E (red triangle) GFAT-1 until 5 mM UDP-GlcNAc (mean ±SD, n=4). **d**, Fo-Fc omit map (green) of UDP-GlcNAc binding to GFAT-1 G451E at a contour level of 3.0 RMSD. A close-up of the UDP-GlcNAc binding pocket with local side chain movements is shown. Proteins are presented as cartoons. UDP-GlcNAc-bound GFAT-1 G451E is depicted in orange and UDP-GlcNAc-free GFAT-1 is colored in gray. UDP-GlcNAc (white sticks) is highlighted. **e**, L-Gln kinetic of Q307A (blue triangle) and Q307A/G451E (green square) GFAT-1 (mean ±SEM, n=3). **f**, Frc6P kinetic of Q307A (blue triangle) and Q307A/G451E (green square) GFAT-1 (mean ±SEM, n=3). Source data are provided as a Source Data file.

Fig. 6 Supp: The G451E substitution activates GFAT-1 in mammalian cells

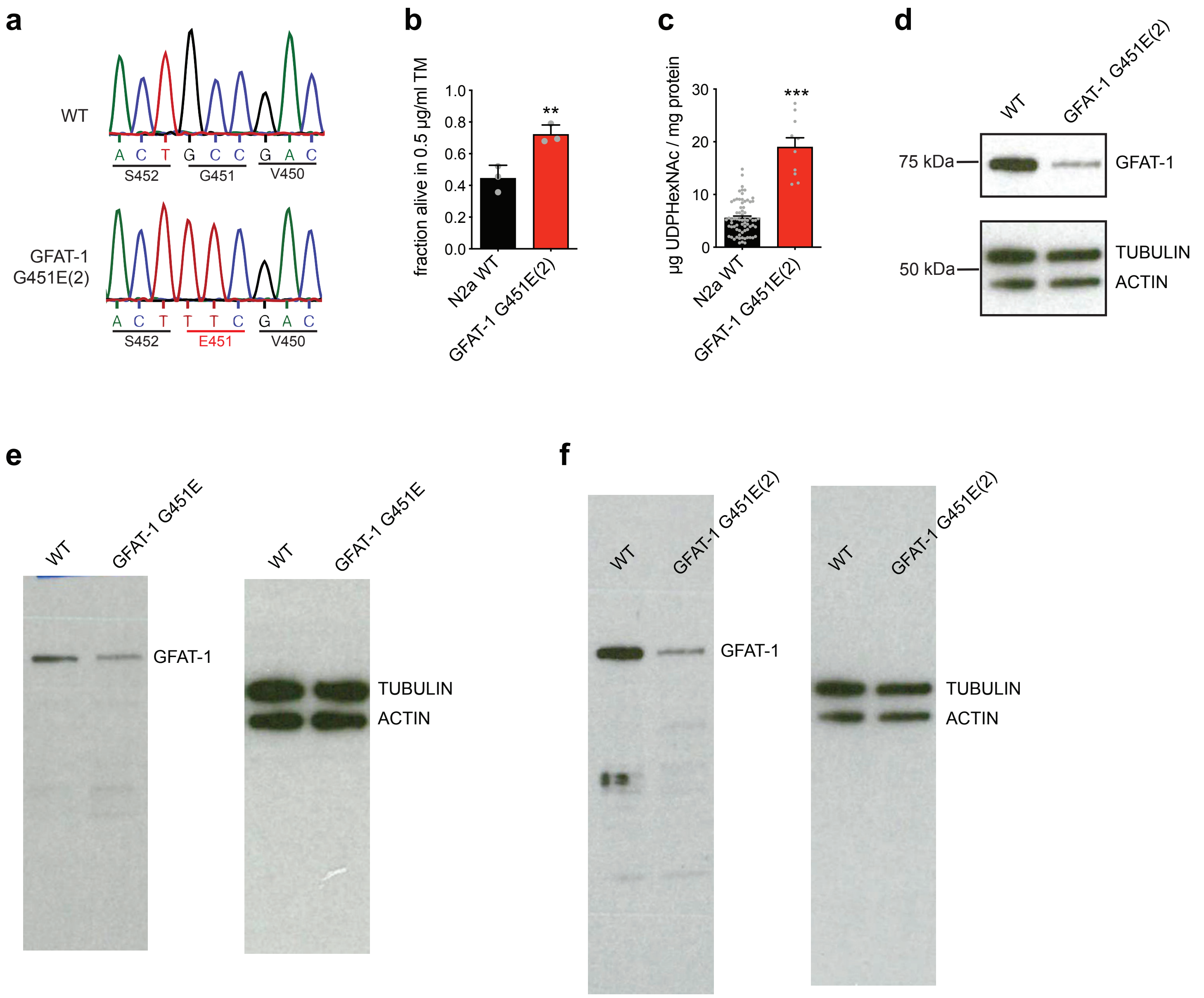

Supplementary Fig. 6: The G451E substitution activates GFAT-1 in mammalian cells.

a, Sanger sequencing results of wild type (WT) and G451E genomic *Gfpt1* engineered in mouse neuroblastoma cells. **b**, Cell viability (XTT assay) of wild type (WT) N2a cells and an independent GFAT-1 G451E cell line (GFAT-1 G451E(2)) after a 48 h treatment with 0.5 μg/ml tunicamycin (TM, mean +SEM, n=3, **p<0.01, unpaired t-test). **c**, UDP-HexNAc level in WT and the GFAT-1 G451E(2) cells (mean +SEM, n≥10, ***p<0.001, unpaired t-test). **d**, Representative Western blot of endogenous GFAT-1 protein from WT cells and the independent GFAT-1 G451E(2) N2a cell line. **e**, **f**, Full Western blots of endogenous GFAT-1 protein levels from WT and GFAT-1 G451E N2a cells. Western blot (**e**) corresponds to cell line GFAT-1 G451E (Fig. 7e) and Western blot (**f**) corresponds to cell line GFAT-1 G451E(2) (Supplementary Fig. 6d). Source data are provided as a Source Data file.
